# The Treatment of Diphtheria in the Past Eight Years

**Published:** 1882-06-20

**Authors:** 


### THE TREATMENT OF DIPHTHERIA IN THE PAST EIGHT YEARS.

Diphtheria has been of late one of the most written-up diseases in medicine. The diligent reader comes to regard the perennial flow of articles on the subject as a sad necessity of progress.

Dr. Ernest Kormann, of Coburg, has done a work which is better than original. It is a review of the literature concerning the treatment of diphtheria in the past eight years. The number of contributions to this branch of the subject alone is very large, being nearly one hundred and seventy. It may be a source of gratification to know that Americans were authors of about one-eighth of these articles. The English contributions numbered only nine, and the French about the same.

It would be impossible to give here any adequate summary of the many views expressed in these numerous papers, but some idea of their general tenor would be, we think, of interest.

There are articles by twenty-four authors on the prophylaxis of diphtheria. These recommend various measures, most of which are known. The most complete harmony is on the subject of isolation and cleanliness. The next most unanimous recommendation is as to the value of frequent gargling. The agents oftenest recommended are potassium chlorate and lime-water. But many recommend salicylic acid, potassium, permanganate, astringents, myrrh, hot and cold water, carbolic acid, etc. Pencilling the throat with carbolic acid or other disinfectants is also advised. Internal medicines receive fewer recommendations except in the case of infants who cannot gargle. Potassium chlorate, salicylic acid, iron, quinine, alcohol, are among the agents enumerated as of use.

Upon the treatment of diphtheria proper there are one hundred and twenty-five contributions, with eighteen more upon the abortive treatment, making one hundred and forty-two in all. This represents a great deal of futile activity; but fortunatcly science is helped by failures as well as success.

A notable feature in the various contributions is that, no matter what the agent recommended, it is almost always an extremely efficient one. The literature of the therapeutics of diphtheria is essentially constructive. Indeed it is too much so, and so judicious iconoclasm would be very useful.

It is generally thought that when a disease has many drugs which are almost its specific, the real specific is nature. But Dr. Kormann suggests that this does not represent the whole truth for diphtheria. That disease may be in some cases or localities very favorably influenced by a special remedy which is of less value at other times or places. However this may be, it is certain that no heterogeneity of remedial measures recommended will justify a physician in adopting an expectant plan of treatment in the disease in question.

And indeed, the diversity in modes of treatment is not fundamentally at all great.

We find that two authors recommend flowers of sulphur blown into the throat. Thirty-eight authors recommend the use of some disinfectant as the essential thing.

Twelve give especial prominence to potassium chlorate; five to salts of iron; thirteen to mecury; four to local applications of chloral hydrate : four to alcohol and other stimulants ; ten to the volatile oils and balsams (turpentine, eucalyptol, copaiva); twenty-six to pilocarpin; two to the application of digestive ferments.

There is the largest number of contributions, as well as the greatest weight of authority in favor of some form of antiseptic and roborant treatment.

The disinfectants recommended most are carbolic, salicylic, and boracic acids. Permanganate of potassium, bromine, chlorine water, ozone, are also mentioned.

But whether disinfectants are recommended or not, chlorate of potash is the agent oftentimes referred to in treatment. The tincture of iron, so extensively used in America, receives less notice among the German writers.

The use of pilocarpine is fully discussed. On the whole, it seems to be a failure. No one has gotten the result first claimed for it. It is not a specific, and several cases of collapse resulting apparently from its use are reported.

The cases illustrating the action of the mercury salts (cyanide, bichloride, etc.,) are in some cases striking, but are too few for a satisfactory conclusion to be drawn regarding them. The methods of treatment by inhalation of oxygen, the use of digestive juices, fluoric acid, lime-juice, chloral, the balsams, etc., as yet have proved little for themselves.

The abortive methods of treatment consist in the careful and complete removal of the first sign of diphtheritic exudation; the very frequent spraying of the throat with weak solutions of salicylic acid, borac acid, or of brandy. The inhalation of steam with gargles of hot water, the local application of strong astringents, or of caustics, and the use of very large doses of alcohol, all of these various measures received about equal commendation.

It is apparent from a study of the literature which we have here referred to, that diphtheria has no specific, nor is it likely to get one, until we find something which will cure all septic diseases. But, there has been progress made in the therapeutics of the disease, and an intelligent physician can save many lives by judicious treatment.—*N. Y. Med. Rec.*

